# Comparative genomics of four strains of the edible brown alga, *Cladosiphon okamuranus*

**DOI:** 10.1186/s12864-020-06792-8

**Published:** 2020-06-26

**Authors:** Koki Nishitsuji, Asuka Arimoto, Yoshitaka Yonashiro, Kanako Hisata, Manabu Fujie, Mayumi Kawamitsu, Eiichi Shoguchi, Noriyuki Satoh

**Affiliations:** 1 Marine Genomics Unit, Okinawa Institute of Science and Technology Graduate University, Onna, Okinawa, 904-0495 Japan; 2 Present address: Marine Biological Laboratory, Graduate School of Integrated Sciences for Life, Hiroshima University, Onomichi, Hiroshima, 722-0073 Japan; 3 Okinawa Prefectural Fisheries Research and Extension Center, Itoman, Okinawa, 901-0354 Japan; 4 DNA Sequencing Section, Okinawa Institute of Science and Technology Graduate University, Onna, Okinawa, 904-0495 Japan

**Keywords:** Genome decoding, *Cladosiphon* strains, Sets of genes, Sub-speciation, Aquaculture, Pan-genome

## Abstract

**Background:**

The brown alga, *Cladosiphon okamuranus* (Okinawa mozuku), is one of the most important edible seaweeds, and it is cultivated for market primarily in Okinawa, Japan. Four strains, denominated S, K, O, and C, with distinctively different morphologies, have been cultivated commercially since the early 2000s. We previously reported a draft genome of the S-strain. To facilitate studies of seaweed biology for future aquaculture, we here decoded and analyzed genomes of the other three strains (K, O, and C).

**Results:**

Here we improved the genome of the S-strain (ver. 2, 130 Mbp, 12,999 genes), and decoded the K-strain (135 Mbp, 12,511 genes), the O-strain (140 Mbp, 12,548 genes), and the C-strain (143 Mbp, 12,182 genes). Molecular phylogenies, using mitochondrial and nuclear genes, showed that the S-strain diverged first, followed by the K-strain, and most recently the C- and O-strains. Comparisons of genome architecture among the four strains document the frequent occurrence of inversions. In addition to gene acquisitions and losses, the S-, K-, O-, and C-strains possess 457, 344, 367, and 262 gene families unique to each strain, respectively. Comprehensive Blast searches showed that most genes have no sequence similarity to any entries in the non-redundant protein sequence database, although GO annotation suggested that they likely function in relation to molecular and biological processes and cellular components.

**Conclusions:**

Our study compares the genomes of four strains of *C. okamuranus* and examines their phylogenetic relationships. Due to global environmental changes, including temperature increases, acidification, and pollution, brown algal aquaculture is facing critical challenges. Genomic and phylogenetic information reported by the present research provides useful tools for isolation of novel strains.

## Background

Brown algae are not only significant primary producers of marine ecosystems, but also have been used as a food resource since ancient times. Recently, they have been cultivated commercially for this purpose. In Japan, the majority of edible brown algae (the class Phaeophyceae) include members of the order Laminariales, *Saccharina japonica* (“kombu” in Japanese) and *Undaria pinnatifida* (“wakame”), and the order Chordariales, *Cladosiphon okamuranus* (“Okinawa mozuku”) and *Nemacystus decipiens* (“ito-mozuku”). Especially in Okinawa (the southwestern prefecture of Japan), *C*. *okamuranus* and *N. decipiens* have been farmed since the 1980s and 1990s, respectively. Approximately 17,000 and 800 tons of these two species were produced in fiscal year 2017. In addition, *C. okamuranus* and *N. decipiens* are sources of fucoidan [1], a sulfated polysaccharide that has anti-coagulant, anti-thrombin-like, and tumor-suppressant activities [2].

For the last three or four decades, global environmental conditions have changed drastically, mainly due to human activities. Greenhouse gas emissions are warming and acidifying the oceans. Pollution from agriculture and sewage is degrading seawater quality, and such problems pose a greater threat to coral reefs in transparent seawater. Aquaculture of “Okinawa mozuku” and “ito-mozuku” in Okinawa has been carried out along the coast, close to coral reefs. Accordingly, brown-alga aquaculture has also been threatened in recent years.

**Fig. 1:**
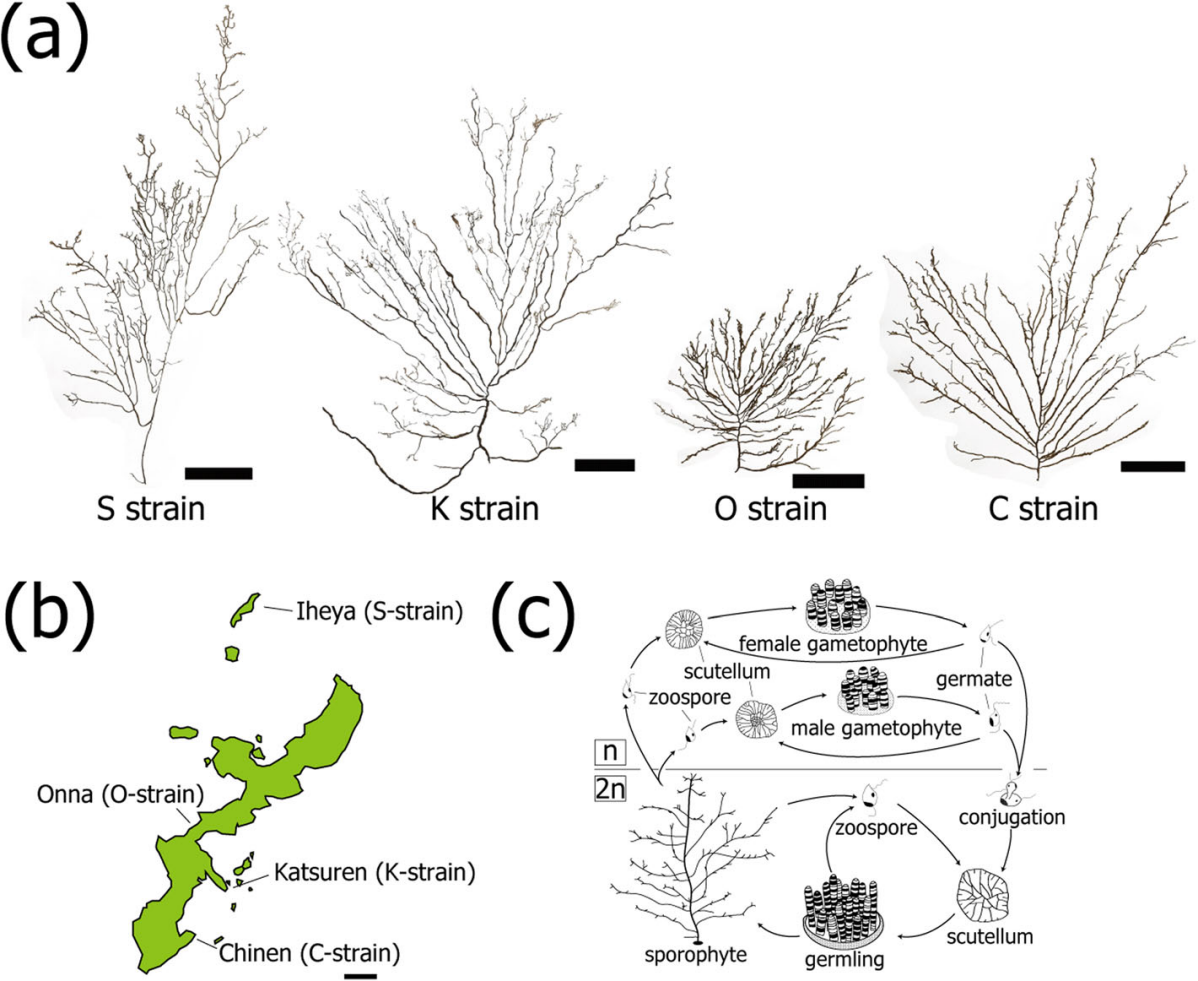
**a** Four strains (S, K, O and C) of *Cladosiphon okamuranus* have distinctive morphologies. Scale bars, 5 cm. **b** Locations from which the four strains were isolated since 2008 in Okinawa, Japan. Scale bar, 10 km. **c** A diagram showing the life cycle of *C. okamuranus*. This alga has n and 2n generations. *C. okamuranus* is cultivated and sporophytes are harvested for market. Genomic DNA was extracted from 2n germlings, while RNA was extracted from 2n germlings and 2n sporophytes.

Given the environmental threats facing phaeophyte aquaculture, it is essential to identify and maintain strains with different physiological features. As mentioned above, culturing of *C*. *okamuranus* commenced in the 1980s and the predominant strain is the S-strain (‘Shikenjo-kabu’, registered as Inou-no-Megumi in Japanese). *C*. *okamuranus* has frond-like sporophytes, the main axes of which are 2 ~ 5 mm in diameter (Fig. 1a). The S-strain exhibits comparatively long lateral branches, and the body is not tough or fibrous.

Given that algae share little similarity with other organisms, the limited amount of genomic data available makes it difficult to determine functions of unknown algal genes [3]. Although nucleic acid extraction from algae is complicated because of the abundance of polysaccharides in cell walls and in the extracellular matrix, we decoded a draft genome of the S-strain in 2016 [4]. During the past 35 years, three other strains have been cultivated, including the K-strain, originally from the Katsuren coast, the O-strain from the Onna coast, and the C-strain from the Chinen coast (Fig. 1b; Supplementary Figure S1). The K-strain comprises thicker, tougher lateral branches (Fig. 1a). The O-strain is composed of smaller, denser lateral branches (Fig. 1a), and the C-strain is intermediate in size, with thinner lateral branches (Fig. 1a). Genetic characterization of the strains is essential for future aquaculture. To help support this industry, we decoded draft genomes of the S- (ver. 2), K-, O- and C-strains.

## Results

### Genome constituents of four strains

The draft genome of *Cladosiphon okamuranus* (S-strain, ver. 1) has been reported previously [4]. The approximately 140-Mbp genome was estimated to contain 13,460 protein-coding genes. The S-strain genome assembly and annotation was improved bioinformatically in this study to approximately 130 Mbp with 12,999 predicted genes (S-strain ver. 2). Nearly 93% of gene models were supported by corresponding mRNAs. Repetitive sequences comprised 11.2% of the genome (Table 1). Quality scores of this newly assembled genome are comparable to those of *Nemacystus decipiens* [5], *Saccharina japonica* [6] and *Ectocarpus siliculosus* [7, 8]*,* although it is difficult to compare these assemblies directly due to the different methods used for each species.

**Table 1:** Comparison of draft genome assemblies of four species of brown algae

Species	*Cladosiphon okamuranus*				*Nemacystus decipiens*^c^	*Ectocarpus siliculosus*^d^	*Saccharina japonica*^e^
strain	S^b^	K^a^	O^a^	C^a^			
Assembled genome size (Mb)	130	135	140	143	154	197	537
No. of scaffolds	541	532	631	291	685	30	13,327
N50 Scaffold (kb)	416	816	752	1051	1863	6528	252
Number of contigs	31,858	5803	5915	6950	411,597	–	29,670
N50 contig size (bp)	21,705	52,668	28,060	44,571	6265	–	58,867
No of genes	12,999	12,511	12,548	12,182	15,156	17,380	18,733
Average gene length (bp)	7949	8430	8817	8636	7902	7542	9587
Average number of introns per genes	9.14	9.51	9.64	9.56	10.24	6.96	–
Average intron length (bp)	530	557	578	579	588	740	–
Repeated sequences (%)	11.2	9.86	11.53	12.58	8.8	22.7	10.57
GC (%)	54	54	54	54	56	54	50
Cegma Completeness (%)	84.3	86.3	86.7	86.7	84.3	72.6	45.6
Cegma Partial (%)	91.9	93.6	94.4	94.4	93.6	87.5	79
Assembler	Newbler	Newbler	Newbler	Platanus	Platanus	–	–

Four strains of *Cladosiphon okamuranus* are classified as the order Chordariales; *Nemacystus decipiens*, the order Spermatochnaceae; *Ectocarpus siliculosus*, the order Ectocarpales; and *Saccharina japonica*, the order Laminariales

^a^The present study; ^b^Nishitsuji et al., [4]; ^c^Nishitsuji et al., [5]; ^d^Cormier et al., [6]; ^e^Ye et al., [7]

Using Illumina platforms, we sequenced and assembled draft genomes of the K-, O-, and C-strains (Supplementary Table S1), which are summarized in Table 1 and Supplementary Figure S2. Scaffold N50s of the four genomes ranged from 416 ~ 1051 kb (Table 1). The GC content of the four strains was identical, amounting to 54% of the DNA sequences (Table 1). The genome size of the K-strain was 135 Mbp, with an estimated 12,511 protein-coding genes. That of the O-strain was 140 Mbp with an estimated 12,548 genes, and that of the C-strain was 143 Mbp with 12,182 estimated genes (Table 1). CEGMA [9] completeness and partial scores were 86 and 94% (Table 1), indicating that genome assemblies of the four strains were adequate for comparative analyses of their genomic and gene constituents.

Repetitive sequences were estimated to constitute 11.2, 9.9, 11.5, and 12.6% of the S-, K-, O-, and C-strain genomes, respectively. This suggests that differences in genome size are not always associated with the number of repetitive sequences, since the smaller S-strain genome (130 Mbp) contained 11.2% repetitive sequences in contrast to the mid-sized K-strain genome (135 Mbp) genome with 9.9% repetitive sequences (Table 1).

DNA transposons comprised 0.4–0.6% of total genome sequences, and RNA transposons comprised an additional 0.6–0.8% (Table 2). Simple repeats and unclassified repeats accounted for 2.2–2.5% and 4.7–5.6% of the genome sequences, respectively. Since the total number of repetitive sequences was larger in the O- and C-strains than in the K-strain, the proportion of each class of repetitive sequences was also larger. No increase or decrease of repetitive sequences was specific to a given strain.

**Table 2:** Classified repeat sequences in the three *Cladosiphon okamuranus* strain genome

Percentage in the assembly						
Genome size			S-strain 135Mb	K-strain 135Mb	O-strain 130Mbp	C-strain 143Mbp
Class of transposons						
DNA transposons						
		hAT-Charlie	0.013%^a^	0.013%	0.012%	0.013%
		TcMar-Tigger	0.000%	0.000%	0.000%	0.000%
	Total		0.547%	0.405%	0.515%	0.551%
Retrotransposons						
LTR						
		ERVL	0.000%	0.000%	0.000%	0.000%
		ERVL-MaLRs	0.000%	0.000%	0.000%	0.000%
		ERV_classI	0.007%	0.007%	0.006%	0.007%
		ERV_classII	0.004%	0.005%	0.004%	0.004%
	Total		2.082%	1.466%	2.263%	2.841%
LINE						
		LINE1	0.006%	0.006%	0.006%	0.006%
		LINE2	0.002%	0.002%	0.002%	0.002%
		L3/CR1	0.018%	0.013%	0.032%	0.030%
	Total		0.749%	0.554%	0.656%	0.795%
SINE			0.009%	0.009%	0.008%	0.008%
Low complexity			0.214%	0.210%	0.209%	0.182%
Simple repeats			2.719%	2.480%	2.442%	2.200%
Satellite			0.030%	0.027%	0.029%	0.023%
Unclassified			5.547%	4.719%	5.416%	5.633%

^a^Percentage in assembled genomes

### Molecular phylogeny of the four strains

All four strains, which were isolated in the early 2000s, have been maintained continuously at the Okinawa Prefectural Fisheries Research and Extension Center (OPFREC). Although the four strains each show unique morphology, the origins of these differences and phylogenetic relationships of the strains are unclear. In order to examine their evolutionary history, we investigated molecular phylogeny using sequences of 32 mitochondrial genes of 41 brown algae and randomly selected 200 single-copy orthologous nuclear genes of six brown algae.

**Fig. 2:**
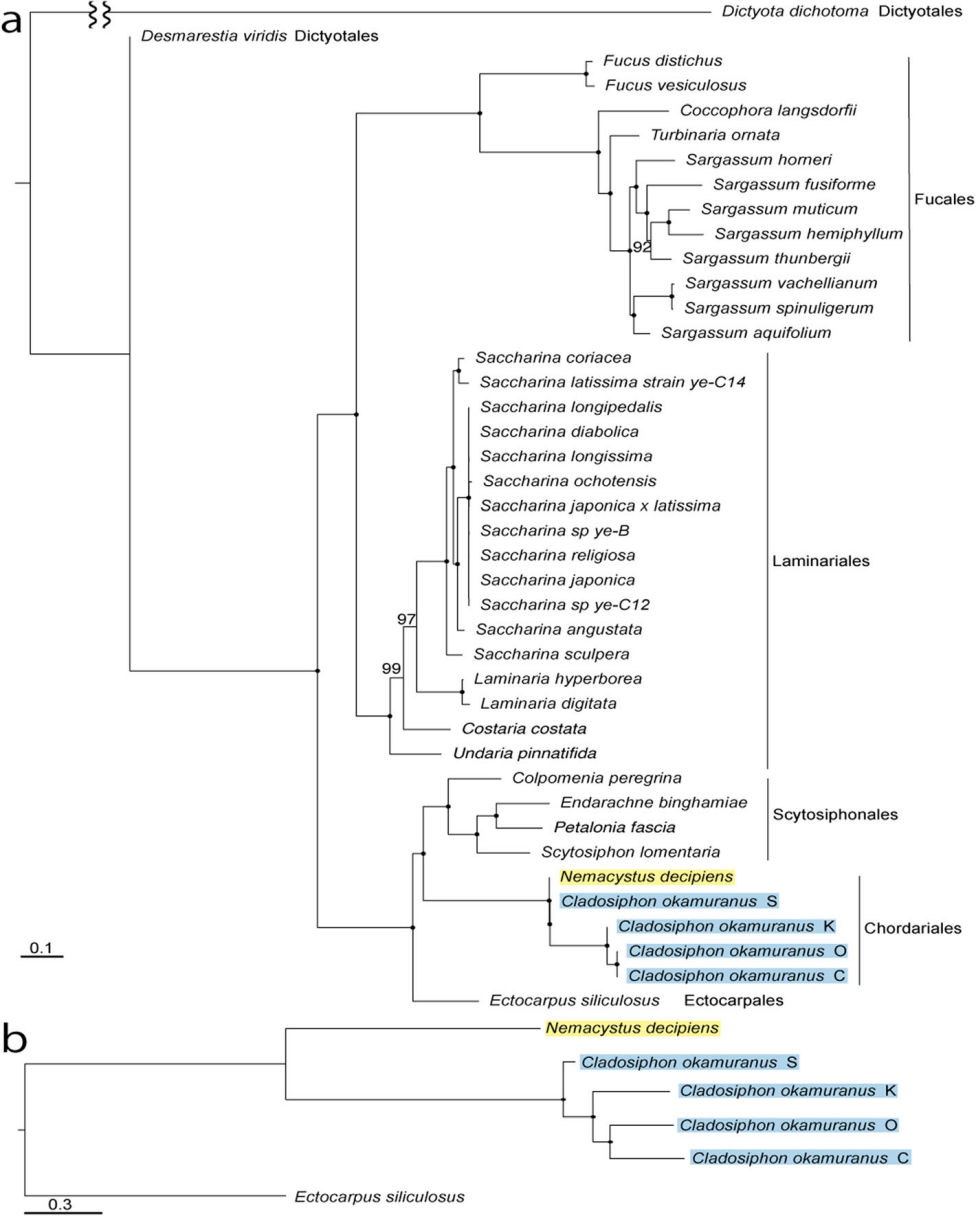
Phylogenetic relationships of the four strains of *Cladosiphon okamuranus*. **a** A molecular phylogenetic tree was constructed using 32 mitochondrial genes and the ML method. *Dictyota dichotoma* was used as an outgroup. Scale bar, 0.1 substitutions/site. **b** A molecular phylogenetic tree was constructed using 200 randomly selected, single-copy, orthologous genes and the ML method. *Ectocarpus siliculosus* was used as an outgroup. Scale bar, 0.3 substitutions/site. Nodes, shown by dots, had 100% bootstrap support (1000 replications), except for nodes with exact numbers.

As was evident in the resulting tree, *Nemacystus* and *Cladosiphon* form a distinct clade, corresponding to the order Chordariales (Fig. 2a, b). In addition, *Cladosiphon okamuranus* constitutes its own distinct clade. The S-strain diverged first, followed by the K-strain, and finally the C- and O-strains. All nodes within the order Chordariales were supported by 100% bootstrap values. The branch length or divergence time between the S-strain and the K/C/O-strains is longer than that between the K-strain and the C/O-strains. The branch length between the C- and O-strains was very short. This suggests that the S-strain is the likely ancestor of the four strains, and that the K-strain probably blanched out first from the K/C/O-strains ancestor. The C- and O-strains are likely the most recently developed.

### Conservation of genome architecture among the four strains

**Fig. 3:**
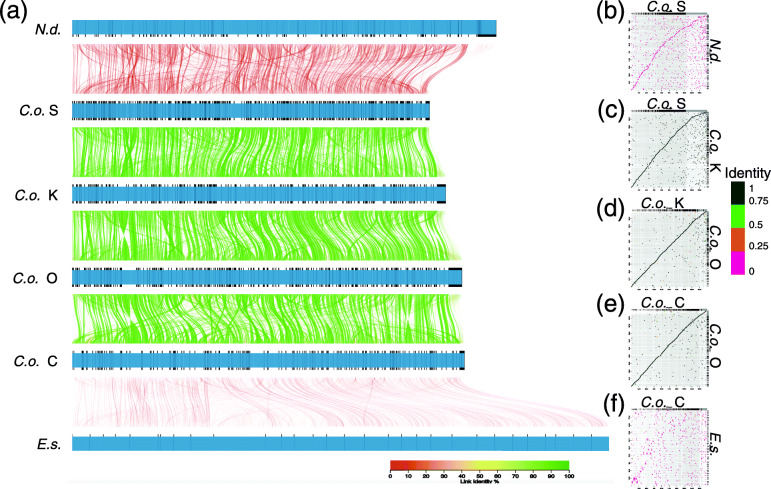
Comparison of genomic architecture in *Nemacystus decipiens*, four *Cladosiphon okamuranus* strains, and *Ectocarpus siliculosus*. **a** Genomic architecture of six brown algal genome sequences. Line color represents the percentage of linked sequence identity. **b**-**f** Dot-plot analysis between *N. decipiens* and *C. okamuranus* S-strain, S- and K-strains, K- and O-strains, O- and C-strains, and *C. okamuranus* C-strain and *E. siliculosus.*

The results of AliTV [10] (Fig. 3a) and Dot-plot analysis using D-Genies [11] (Fig. 3b-f) suggested that the four strains of *C. okamuranus* exhibit similarities exceeding 90% for each comparison. Genome-wide sequence similarity was not so high between the *C. okamuranus* S-strain and *N. decipiens* (compare the upper lane with next lane), and it was low between the *C. okamuranus* C-strain and *E. siliculosus*.

A similar profile of genome-wide sequence resemblance was evident in the Dot-plot analysis (Fig. 3b-f). Linearity of dot plots was evident between the S- and K-strain (Fig. 3c), between the K- and O-strain (Fig. 3d), and between the C- and O-strain (Fig. 3e). On the other hand, a weaker linear correlation was evident in sequence comparisons between the S-strain and *N. decipiens* (Fig. 3b), and almost no relationship exists between C-strain and *E. siliculosus* (Fig. 3f). The overall similarity was ~ 50% between the *C. okamuranus* S-strain and *N. decipiens* (Fig. 3a, b), and less than 7% between the *C. okamuranus* C-strain and *E. siliculosus* (Fig. 3a, f). *C*. *okamuranus* belongs to the family Chordariaceae of the order Chordariales, whereas *N. decipiens* pertains to the family Spermatochnaceae in the same order. *E. siliculosus* belongs to a different order, the Ectocarpales. Differences in genome-wide sequence similarity may depend on time since divergence, during which neutral DNA-sequence changes likely occurred.

Inversions appear to have occurred during diversification of the four strains, especially between the O- and C-strains (Fig. 3a). Although results from AliTV and Dot-plot analysis cannot be directly compared, the dot-plot profiles of O- and C-strains suggested similar frequencies of inversions.

### Synteny analysis of the four strain genomes

**Fig. 4:**
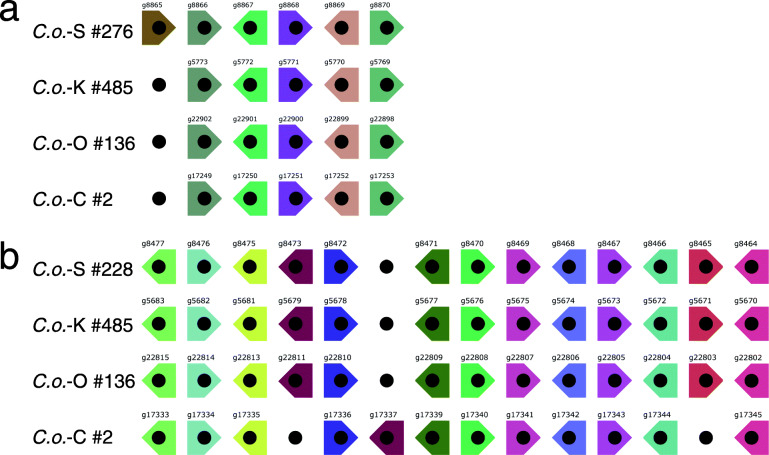
Two examples of synteny blocks among genomes of the four strains. **a** A block comprising six genes. Orthologous relationships are indicated by colors. Gene IDs are shown above each block. **b** Another block composed of 13 genes. Orthologous relationships are indicated by colors. One gene inversion and one gene deletion are evident in the C-strain. Scaffold numbers are shown to the left of rows. Arrowheads indicate gene directions

Next we examined synteny of genes in the four *C*. *okamuranus* genomes*.* Analyses using i-ADHoRe [12] identified 550 genomic regions showing shared synteny among the four genomes (Fig. 4). For example, scaffold #276 of the S-strain manifests a syntenic region comprising six genes (gene IDs, g8865, g8866, g8867, g8868, g8869 and g8870) (Fig. 4a). The K-, O-, and C-strains each retained scaffolds corresponding to S-#276, (K-#485, O-#136, and C-#002). In this synteny, g8865 was present only in the S-strain, suggesting a gene loss in the ancestor of the K/O/C strains after their divergence from the S-strain. This provides support for the phylogenetic gap between the S-strain and K/O/C-strains, discussed earlier.

Scaffold #228 of the S-strain offers a second example comprising 13 genes (gene IDs, g8477, g8476, g8475 g8473, g8472, g8471, g8470, g8469, g8468, g8467, g8466, g8465, and g8464) (Fig. 4b). All genes occur in exactly the same order in corresponding scaffolds K-#485 and O-#136, although two differences were evident in the C-strain. One is an inversion of g17337, corresponding to g8473 of the S-strain, moving beyond g17336 and next to 17,339 (Fig. 4b). The other is a lack of g8465 (Fig. 4b). This result provides further support for the supposition that the C-strain genome was uniquely modified after its divergence.

### Analysis of orthologous gene families

**Fig. 5:**
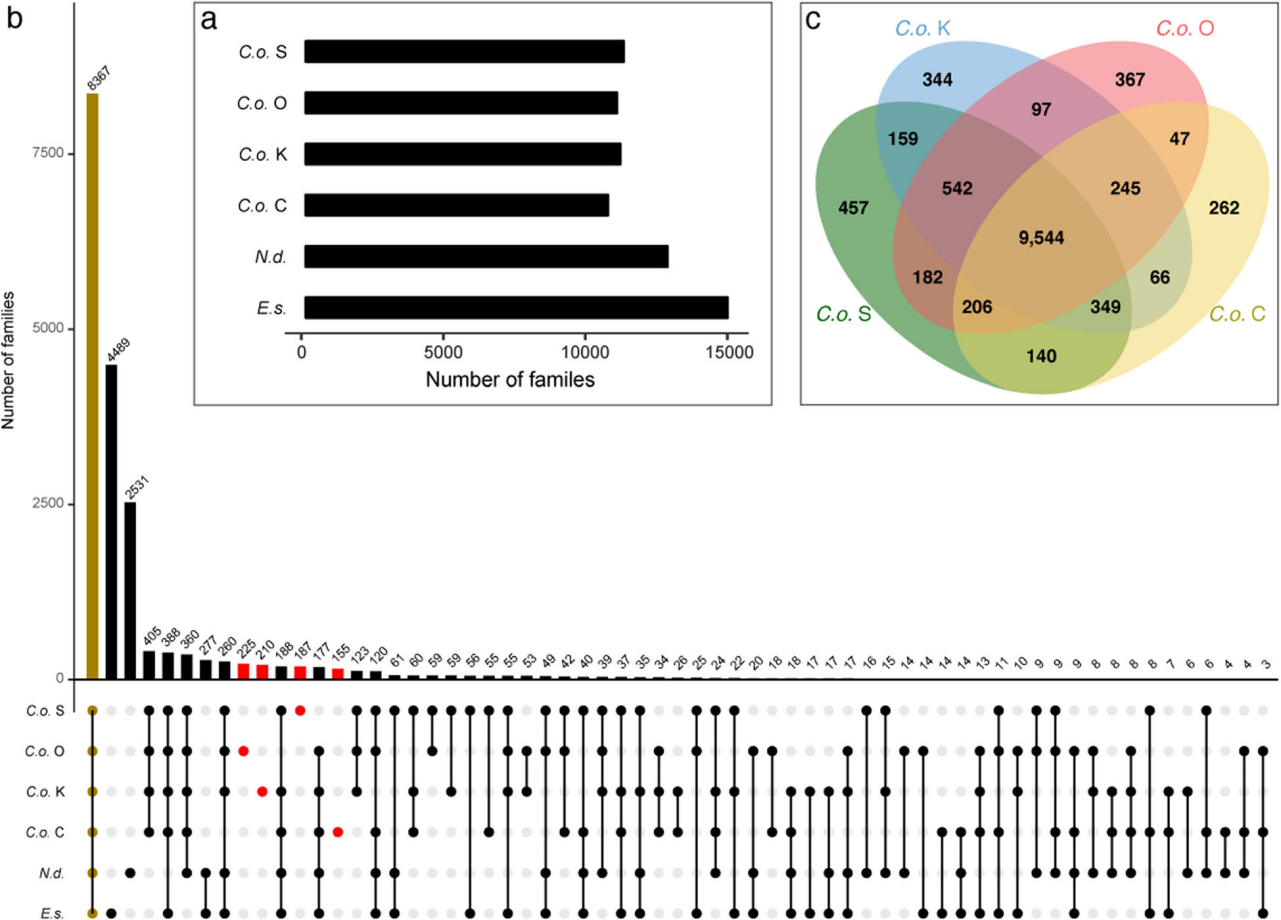
Orthologous gene analysis in genomes of *Cladosiphon okamuranus* S/K/O/C-strain, *Nemacystus decipiens,* and *Ectocarpus siliculosus.***a** Numbers of gene family in the six genomes. **b** Shared families are shown on the horizontal axis and the number of patterns in the vertical axis. 8367 families shared by all the six genomes are shown in gold, while families specific to individual *Cladosiphon* strains are shown in red. The analysis demonstrated the presence of 187, 210, 225, and 155 families unique or specific to the S-, K-, O- and C-strains, respectively. **c** Venn diagram showing the numbers of gene families shared among the four *Cladosiphon okamuranus* strains. Each strain contains a number of unique gene families

The genome project documented approximately 12,500 genes in genomes of the four strains of *Cladosiphon* ([4], the present study), 15,156 genes in the *Nemacystus* genome [5] and 17,418 genes in the *Ectocarpus* genome [6] (Table 1). Orthologous analysis of numbers of gene families in each genome (Fig. 5a) demonstrated that 8367 gene families were shared or conserved by the six genomes (Fig. 5b). On the other hand, 4489 families were unique to *Ectocarpus*, 2532 to *Nemacystus,* and 405 to *Cladosiphon,* respectively. In addition, this analysis demonstrated the presence of unique families in each strain: 187 families unique to the S-strain, 210 to the K-strain, 225 to the O-strain, and 155 to the C-strain (Fig. 5b). There were many patterns depending on how families were shared by combinations of the six genomes (Fig. 5b). For example, different numbers of gene families were shared among different combinations of strains: 123 (S/O/K), 60 (S/K/C), 59 (S/O), 59 (S/K), 55 (S/C), 53 (O/K), 42 (S/O/C), 34 (O/K/C), 26 (K/C), and 18 (O/C), providing more information about diversification of the four strains.

We further compared gene families in the four *Cladosiphon* strains using OrthoFinder [13]. The four strains shared 9544 gene families (Fig. 5c), but the S-, K-, O-, and C-strains include 457, 344, 367, and 262 unique families each, constituting 3.5, 2.7, 2.9, and 2.2% of the orthologous genes in those strains, respectively. These gene families may be involved in the evident morphological diversification of the four strains, and may also support different physiology as well, although that remains to be explored.

**Fig. 6:**
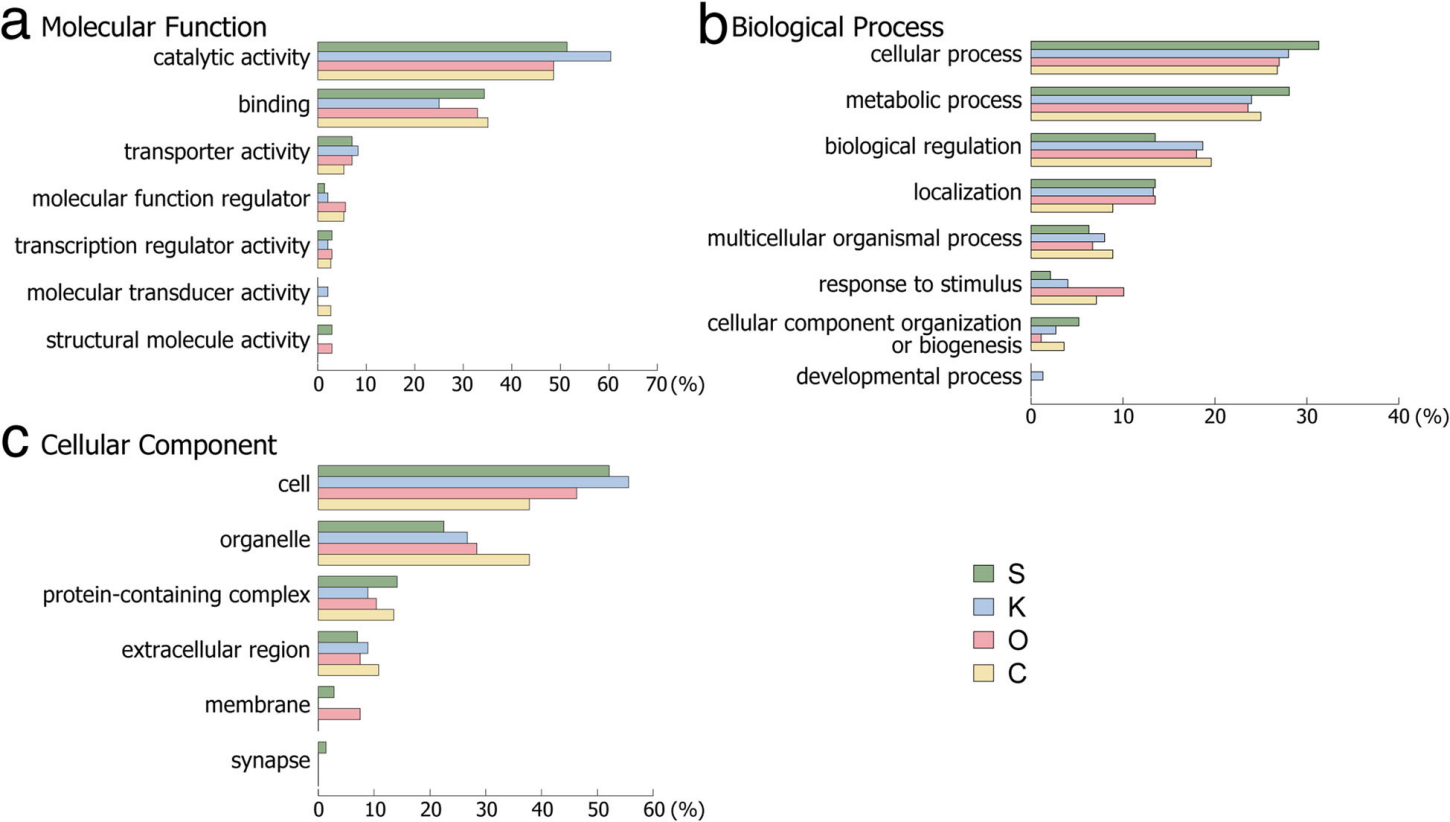
GO annotation of genes with unknown functions that are unique to each *Cladosiphon okamuranus* strain.

It would be highly desirable to know the functions of these unique gene families; however, extensive Blast [14] searches failed to identify orthologies for most of them, meaning that their functions are novel. Therefore, we performed GO annotation analysis of these genes (Supplementary Table S2), based on “molecular function” (Fig. 6a), “biological process” (Fig. 6b), and “cellular component” (Fig. 6c). In general, the four strains displayed similar GO annotation profiles. All four contained similar numbers of genes related to “catalytic activity” and “binding” in the category “molecular function” (Fig. 6a), and to “cellular process” and “metabolic process”, subcategories of “biological process” (Fig. 6b), and to “cell and organelle”, under the heading of “cellular component” (Fig. 6c). Some unknown genes were specific to only one or two strains (Fig. 6).

## Discussion

Brown algae have served as a food resource since ancient times. In Japan, especially in Okinawa, *Cladosiphon okamuranus* (“Okinawa mozuku”) has been commercially farmed since the 1980s, yielding approximately 15 kt per year. Four strains of *C. okamuranus* with different morphologies, the S-, K-, O-, and C-strains, have been maintained at the Okinawa Prefecture Fisheries Research and Extension Center since the early 2000s. However, algal aquaculture in Okinawa now faces various threats, mostly due to surface seawater temperature increases and declining seawater quality. In such circumstances, it is desirable to understand genetic properties and the evolutionary trajectories of the strains. Such genic and genomic information may help improve aquaculture methods and/or production of new strains with greater ability to withstand environmental stresses. To this end, we decoded the 130 ~ 143 Mbp nuclear genomes of the four *C. okamuranus* strains. The S-strain genome was first assembled in a previous study [4], but was improved in this study, and the K-, O- and C-strain genomes were assembled for the first time in this study. The quality of all four genomes is comparable to those of other algal genomes.

Molecular phylogeny using mitochondrial and nuclear DNA sequences allowed us to infer the evolutionary trajectories of the four strains (Fig. 2). The S-strain diverged first, then the K-strain, and finally the O- and C-strains. Both analyses, one based on mitochondrial DNA sequences (Fig. 2a) and the other on nuclear DNA sequences (Fig. 2b), resulted in identical tree topologies. In addition, all nodes had 100% bootstrap support, indicating that this represents the most probable history of the four strains. Only one difference was noticed between the two trees. That is, the branch that connects *Nemacystus* and the *Cladosiphon* S-strain was much shorter in the mitochondrial tree than in the nuclear tree. This suggests an occurrence of introgression of mitochondrial genome from *Nemacystus* to the S-strain or common ancestor of *C. okamuranus.* This question should be examined in future studies.

Each strain exhibits different morphology (Fig. 1a). Together with results of molecular phylogeny, we speculate that the morphology of the *Cladosiphon okamuranus* ancestor was likely close to that of the extant S-strain, with comparatively long lateral branches and a soft body, because the closely-related *Nemacystus decipiens* exhibits morphology similar to that of the S-strain. From this ancestor, the K-strain became a middle-sized sporophyte with thicker and tougher lateral branches, and the O-strain diverged further with smaller and more dense lateral branches. The C-strain is intermediate in size with thinner lateral branches (Fig. 1a), respectively. This information appears useful for further isolation of *C. okamuranus* strains, which is ongoing at OPFREC.

Genome architecture of each *Cladosiphon* strain varied from 130 to 143 Mbp. It is generally accepted that there is some degree of correlation between the genome size, the proportion of repetitive sequence, and the number of genes [15, 16]. In *Cladosiphon* however such assumption is not so simple (Table 1). Instead, each of the four genomes has likely undergone its own modification. Although long-read sequence technology might reveal the relationship between the genome size and the repetitive sequence, technical issues like the presence of lots of polysaccharide in DNA samples have impeded to introduce such methods. It remains to establish new methods, by which we make these questions clear in near future.

The most interesting finding was that the four strains contain unique gene families that equate to 2 to 3.5% for all genes (Supplementary Table S2). Such proportions are quite higher than that of seven strains of *Arabidopsis thaliana* (0.79%) [17]. This might suggest that the genetic distances among the four strains of *Cladosiphon* might reach the grade, in which the four strains are distinguished as “related species or subspecies.” Although we conducted BLAST searches of these genes, most of them showed no sequence similarity to any genes in the NCBI database; hence, their functions are unknown. GO annotations for these genes indicated that most of them are associated with biological functions, such as metabolic processes and cellular components. It is likely that these gene families are involved in development of the distinctive morphology of the four *Cladosiphon* strains, although details of molecular mechanisms remain to be explored. It is entirely possible that they also underlie variations in morphology. Our investigation also revealed the occurrence of many inversions in the genomes during their diversification (Figs. 3a; 5b). Although frequent inversion of genomic regions is known in land plants [18], this may be the first report showing this phenomenon in brown algal genomes.

Land plant genomes have been analyzed to support basic biology and applied use as well. Comparative genomics of the deciphered genomes show the expansion of genes that may be required for their survival [19]. Diatoms, like brown algae, are classified as Stramenopiles, and various diatom genomes have been decoded due to their relatively smaller genome sizes. Analyses of diatom genomes suggest the occurrence of horizontal gene transfers from bacteria, and documents a lack of photosynthesis-related genes as well [20, 21]. On the other hand, comparative genomics of brown algae are still limited. Analyses have been restricted to genes related to fucoidan biosynthesis, extracelluar components, stress responses, and vanadium-dependent haloperoxidases [4, 5, 22]. In addition to taxon-level genomics, comparative genomics of strains / subspecies also contribute to our understanding of macroalgal evolution.

## Conclusions

Genome analyses of four *C. okamuranus* strains with distinctively different morphologies revealed that they possess unique gene families, and phylogenetic analysis indicated that the S-strain is closest to the ancestral strain. *C*. *okamuranus* has been cultivated in Okinawa since the 1980s. Approximately 17,000 tons of *C*. *okamuranus* were produced in fiscal year 2017, representing more than 90% of the entire Japanese harvest. Due to global environmental changes, including temperature increases, ocean acidification, and pollution, brown algal aquaculture is facing critical challenges. Efforts to maintain and improve culture methods are required, and heat stress experiments are under way at OPFREC to identify strains that tolerate warmer ocean temperatures. The genomic information reported in this study may help to develop and characterize new strains.

## Methods

### Brown algal strains

Originally, four strains of *Cladosiphon okamuranus* were isolated from four local fisheries (Fig. 1b). The S-strain came from Iheya in 2008, the O-strain from Onna in 2008, the K-strain from Katsuren in 2009, and the C-strain was collected in Chinen in 2012 (Fig. 1b). From 2n clonal cycles, 2n zoospores are isolated from sporophytes, and maintained as 2n germlings on plates. Each strain exhibits characteristic morphology, distinguishing it from the others. Each strain has been maintained as a stock culture at OPFREC, Okinawa, Japan. *Cladosiphon* is cultivated at 22.5 °C with a 12-h light-dark cycle in seawater containing 0.5% KW21 (Daiichi Seimo Co., Ltd., Kumamoto, Japan). The life cycle of *C. okamuranus* includes both haploid (n) and diploid (2n) generations (Fig. 1c). 2n germlings mature into sporophytes that are harvested for market.

### DNA extraction, genome sequencing, and assembly

For DNA extraction, 2n germlings of each strain were frozen in liquid nitrogen and stored at − 80 °C until use. They were crushed to powder with a frozen-cell-crusher, Cryo-Press (Microtec Co., Ltd., Chiba, Japan) and genomic DNA was extracted from the powder using a DNA extraction kit, DNA-Suisui-VS (Rizo Co., Ltd., Ibaraki, Japan). The strains have been maintained as protonemas without contamination from other eukaryotes, allowing us to extract strain-specific, genomic DNA [4].

Illumina MiSeq, HiSeq Rapid, and HiSeq 4000 platforms were used for sequencing [23]. Libraries were prepared with slight protocol modifications recommended by the manufacturer. Fragmented genomic DNA was further purified using Blue Pippin (Sage Science, Beverly, MA, USA). A paired-end library consisting of 700-bp clones was prepared for the MiSeq using a TruSeq DNA PCR-Free LT Sample Prep Kit (Illumina, San Diego, CA, USA), and mate-pair libraries with insert lengths of 2-, 3-, 4-, 5-, 6-, 7-, 8-, 10-, 12-, and 14-kbp were prepared for the HiSeq 4000 using a Nextera Mate Pair Sample Prep Kit (Illumina) (Supplementary Table S1).

K-mer counting and estimation of genome sizes were accomplished with JELLYFISH 2.2.0 [24, 25] and GenomeScope [26]. Adapter sequences were trimmed from all reads using Trimmomatic-0.30 [27]. High-quality, paired-end reads (quality > 20) were assembled de novo using Newbler 2.9 [28] and Platanus 1.2.4 [29] to create contigs. Better assembled sequences were used for downstream analysis. Subsequent scaffolding of the Newbler or Platanus output was performed using SSPACE 3.0 [30], based on Illumina mate-pair information [31]. Gaps inside scaffolds were closed using GapCloser 1.12 [32].

Diploid sequences were merged using HaploMerger2 v3.4 [33] and BLASTN (1e^− 50^) aligned by more than 50%. CEGMA 2.5 was used to evaluate genome assembly. Sequences that likely originated from bacteria and other microbiota were removed from the assembled genome with Maxbin version 2.2 [34] and RNAmmer 1.2 [35]. Paired-end genomic DNA reads that were not used in each of the strain-genome assembly processes were collected with kneaddata v0.6.1 (https://bitbucket.org/biobakery/kneaddata/wiki/Home). Those reads were assembled with novoPlasty (version2.7.2) [36] for the chloroplast and mitochondrial genomes of *C. okamuranus*.

### Transcriptome analyses

RNA was isolated from sporophytes (2–5 cm) and 2n germlings (Fig. 1c). Total RNA was extracted according to manufacturer instructions, using DNase and RNeasy Plant mini kits (QIAGEN, Hilden, Germany). Transcriptome libraries were prepared using a TruSeq Stranded mRNA Library Prep kit (Illumina). RNA was sequenced as per manufacturer instructions for the Illumina HiSeq Rapid and HiSeq 4000. Only sequences of high quality (quality > 20) were assembled, using Velvet 1.2.10 [37] and Oases 0.2.08 [38].

### Transposable elements and repetitive sequences

Tandem repeats were detected and classified using RepeatModeler-1.1.8 (http://www.repeatmasker.org/RepeatModeler/). A de novo repeat library was generated with RepeatScout (version 1.0.5) [39]. Transposons and SINE in the scaffold were identified using RepeatMasker (ver. 4.0.7, http://www.repeatmasker.org/RMDownload.html) with the Repbase (version 21.01) [40].

### Gene model prediction

For each strain genome, a set of gene model predictions (*C. okamuranus* Gene Model ver. 1) was generated with AUGUSTUS 3.2.1 [41], which was trained on transcriptome contigs recommended by PASA 2.2.0 [42]. Gene models were predicted by running AUGUSTUS, by mapping RNA-seq and transcriptome data to the genome, and by using results of repetitive sequence analysis to greatly improve gene prediction accuracy. Predicted gene models were refined with PASA.

### Gene functional annotation

In order to identify putative *C. okamuranus* orthologous genes, reciprocal BLAST analysis was performed. This was carried out using mutual best hits with genes from *N. decipiens*, *Ectocarpus siliculosus*, and the non-redundant protein sequences database from NCBI against *C. okamuranus* gene models (BLASTP) or their assembly (TBLASTN). A second approach used for encoded proteins with one or more specific protein domains was to screen the models using HMMER (hmmer3) [43] against the Pfam database (Pfam-A.hmm, release 24.0, http://pfam.sanger.ac.uk) [44], which contains approximately 11,000 conserved domains. Encoded proteins were also analyzed using InterProScan 5.25–64.0 [45] and the SwissProt database for gene ontology and functional annotation.

### Identification of orthologous gene groups and synteny analysis

Protein sequences of *C. okamuranus*, *N. decipiens*, and *E. siliculosus* were analyzed with OrthoFinder version 2.0.0, using default parameters to identify orthologous gene groups. Synteny of six brown algal genomes was analyzed with i-ADHoRe 3.0 using default parameters.

### Gene collection for phylogenetic tree analysis

Sets of related sequences were subjected to phylogenetic analyses to more precisely determine orthologous relationships among *C. okamuranus* strains. Mitochondrial genome sequences of 37 brown algae were downloaded from the NCBI database or our genome browsers. Mitochondrial genomes were annotated using GeSeq. cDNA sequences of *Atp6*, *Atp8*, *Atp9*, *Cox1*, *Cox3*, *Cob*, *Nad1*, *Nad2*, *Nad3*, *Nad4*, *Nad4l*, *Nad5*, *Nad6*, *Nad7*, *Nad9*, *Rpl2*, *Rpl5*, *Rpl14*, *Rpl16*, *Rpl31*, *Rps2*, *Rps3*, *Rps4*, *Rps7*, *Rps8*, *Rps10*, *Rps11*, *Rps12*, *Rps13*, *Rps14*, *Rps19*, and *Tatc* genes from brown algae were collected. 200 randomly selected, single-copy, orthologous nuclear genes of six brown algae were collected using OrthoFinder output (Supplementary Table S3). 32 mitochondrial and 200 nuclear gene sequences were independently aligned using MAFFT [46] with default options. Spurious sequences or poorly aligned regions were filtered using trimAl [47]. Then filtered sequences were concatenated. Phylogenetic trees were constructed using the maximum likelihood method (GTR-gamma model) with RAxML version 8.2.11 [48] with partition analysis excluding third codon positions and 1000 bootstrap replicates.

### Genome-wide comparative analysis among six brown algal genomes

AliTV was used to examine the overall similarity of six genomes, including four strains of *C. okamuranus*, with *N. decipiens, and E. siliculosus* as outgroups*.* Using D-Genies, Dot-plot analysis was also used to compare sequence similarities between them (15 pairwise comparisons of the six genomes, using a 10-kb sliding window).

## Supplementary information

**Additional file 1: Figure S1.** Location of Okinawa in Japan Okinawa is the southernmost prefecture of Japan. **Figure S2.** A summary of genome size estimates for three strains of *Cladosiphon okamuranus* using GenomeScope.

**Additional file 2: Table S1.** A summary of DNA and RNA sequence data from three strains of *Cladosiphon okamuranus.***Table S2.** Numbers of GO annotated genes in *Cladosiphon okamuranus* strain-specific gene groups. **Table S3.** Gene IDs of randomly selected 200 single-copy orthologous nuclear genes.

## Data Availability

Sequence data were deposited in DDBJ as BioProject ID, PRJDB9346. A genome browser has been established for the assembled genome sequences using the JavaScript-based Genome Browser (JBrowse) 1.11.6 [49]. Assembled sequence and gene models are accessible at http://marinegenomics.oist.jp/gallery/.

## Abbreviations

GO: Gene Ontology
*C.o.*: *Cladosiphon okamuranus*
*N.d.*: *Nemacystus decipiens*
*E.s.*: *Ectocarpus siliculosus*
OPFREC: Okinawa Prefectural Fisheries Research and Extension Center
