# Supplementary material for: Comparative genomics of four strains of the edible brown alga, Cladosiphon okamuranus
Source: BMC Genomics. 2020 Jun 26;21:422. doi: 10.1186/s12864-020-06792-8 (PMC7318753; doi:10.1186/s12864-020-06792-8)
Supplement: Supplementary file 1 — Additional file 1: Figure S1. Location of Okinawa in Japan Okinawa is the southernmost prefecture of Japan. Figure S2. A summary of genome size estimates for three strains of Cladosiphon okamuranus using GenomeScope. [file 12864_2020_6792_MOESM1_ESM.pdf]

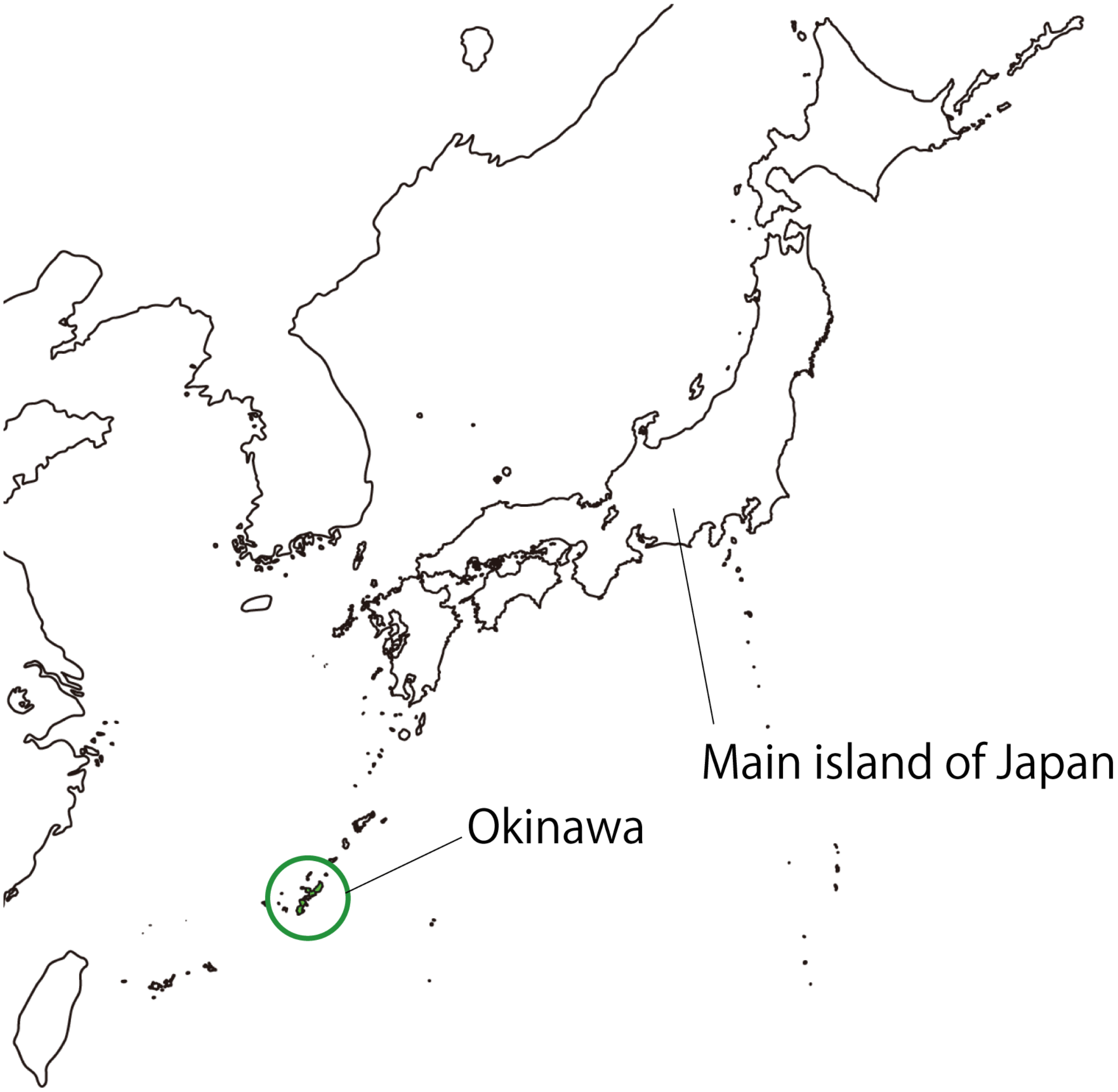

Supplementary Figure S1. Location of Okinawa in Japan

Okinawa is the southernmost prefecture of Japan.

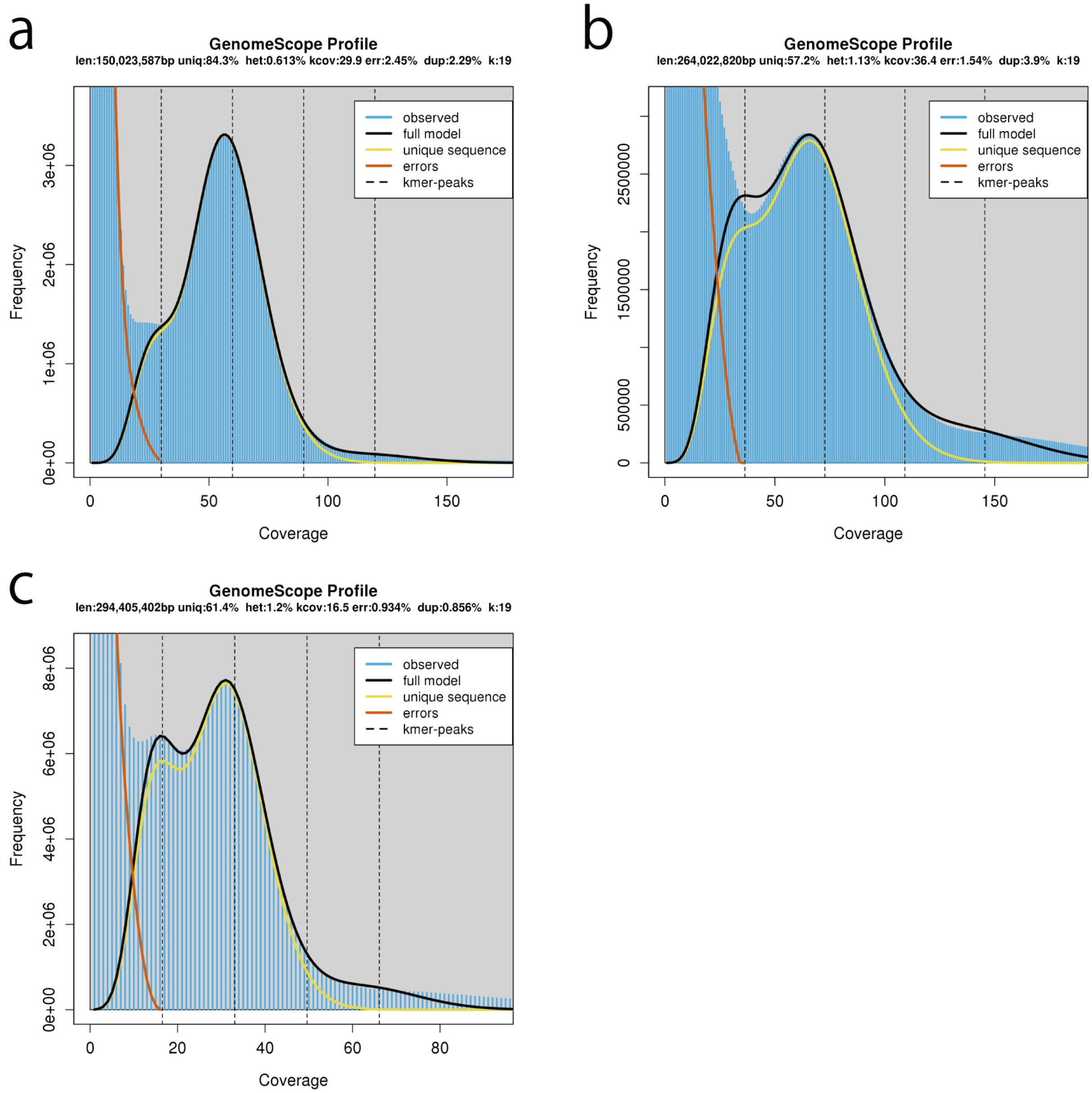

Supplementary Figure S2. A summary of genome size estimates for three strains of *Cladosiphon okamuranus* using GenomeScope

(a) *C. okamuranus* K-strain. (b) *C. okamuranus* O-strain. (c) *C. okamuranus* C-strain. These estimated genome sizes contained both *C. okamuranus* and contaminating bacterial genomes.
